# Complete genome sequence of the sand-sediment actinobacterium *Nocardioides dokdonensis* FR1436^T^

**DOI:** 10.1186/s40793-017-0257-z

**Published:** 2017-07-25

**Authors:** Min-Jung Kwak, Soon-Kyeong Kwon, Jihyun F. Kim

**Affiliations:** 1 Department of Systems Biology and Division of Life Sciences, Yonsei University, 50 Yonsei-ro, Seodaemun-gu, Seoul 03722 Republic of Korea; 2 Strategic Initiative for Microbiomes in Agriculture and Food, Yonsei University, 50 Yonsei-ro, Seodaemun-gu, Seoul 03722 Republic of Korea

**Keywords:** *Nocardioidaceae*, Propionibacteria, Corynebacteria, Cholesterol, Steroid medicine

## Abstract

*Nocardioides dokdonensis*, belonging to the class *Actinobacteria*, was first isolated from sand sediment of a beach in Dokdo, Korea, in 2005. In this study, we determined the genome sequence of FR1436, the type strain of *N. dokdonensis*, and analyzed its gene contents. The genome sequence is the second complete one in the genus *Nocardioides* after that of *Nocardioides* sp. JS614. It is composed of a 4,376,707-bp chromosome with a G + C content of 72.26%. From the genome sequence, 4,104 CDSs, three rRNA operons, 51 tRNAs, and one tmRNA were predicted, and 71.38% of the genes were assigned putative functions. Through the sequence analysis, dozens of genes involved in steroid metabolism, especially its degradation, were detected. Most of the identified genes were located in large gene clusters, which showed high similarities with the gene clusters in *Pimelobacter simplex* VKM Ac-2033D. Genomic features of *N. dokdonensis* associated with steroid catabolism indicate that it could be used for research and application of steroids in science and industry.

**Electronic supplementary material:**

The online version of this article (doi:10.1186/s40793-017-0257-z) contains supplementary material, which is available to authorized users.

## Introduction

Bacteria in the genus *Nocardioides* were first isolated from soil in 1976 [1] and currently more than 90 validly published *Nocardioides* species are available from diverse terrestrial and aquatic environments such as soil, wastewater, plant roots, groundwater, beach sand, and marine sediment [2–10]. Originally, the genus was classified as a member of the order *Actinomycetales* in the phylum *Actinobacteria*, but recently was reclassified to the order *Propionibacteriales* [11]. *Actinobacteria*, also called Gram-positive high G + C bacteria, contain diverse bacterial groups that are capable of a variety of secondary metabolism including biosynthesis of antibiotics and degradation of harmful compounds [12, 13]. The genus *Nocardioides* is also known to utilize several kinds of non-degradable materials such as alkane compounds [14], atrazine [15], phenanthrene [16], trinitrophenol [17], and vinyl chloride [18]. Despite almost 100 species with validly published names and their useful features associated with secondary metabolism, only draft genome sequences are publically available for the genus besides that of *Nocardioides* sp. JS614.

*N. dokdonensis* was isolated from beach sand in Dokdo, a volcanic island located in the East Sea of Korea, in 2005 [19]. The East Sea is called a “mini-ocean” due to its oceanological properties [20] and is known to have a high microbial diversity [21]. To reveal distinguishing genomic features of *Nocardioides* species, we determined and analyzed the genome sequence of *N. dokdonensis* FR1436^T^.

## Organism information

### Classification and features

**Fig. 1:**
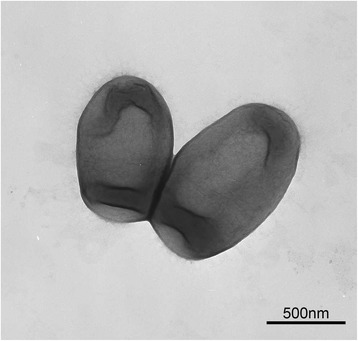
Transmission electron microscopic image of *N. dokdonensis* FR1436

*Nocardioides dokdonensis* FR1436^T^, a Gram-positive, non-motile, and strictly aerobic bacterium, was isolated from sand sediment of the Dokdo island in Korea [19]. The strain grows at the temperature range of 4 to 30 °C (optimum, 25 °C), pH range of 5.0 to 10.0 (optimum, 7.0), and NaCl concentration of 0 to 7% (*w*/*v*) (optimum, 0 to 3) [19]. Its colony size is about 1.0–2.0 mm on TSA medium after incubation for 3 days at 25 °C. Cells are 1.2–1.8 μm long and 0.6–0.9 μm wide in size [19] (Fig. 1). FR1436 can utilize adonitol, glycerol, melezitose, melibiose, ribose, sodium acetate, sodium citrate, sodium propionate, and sodium pyruvate as a sole carbon source [19]. Minimum information about the genome sequence (MIGS) for FR1436 is described in Table 1.

**Table 1:** Classification and general features of *N. dokdonensis* FR1436 according to the MIGS recommendations [39]

MIGS ID	Property	Term	Evidence code^a^
	Classification	Domain *Bacteria*	TAS [40]
		Phylum *Actinobacteria*	TAS [41]
		Class *Actinobacteria*	TAS [42]
		Order *Propionibacteriales*	TAS [11]
		Family *Nocardioidaceae*	TAS [11]
		Genus *Nocardioides*	TAS [43]
		Species *Nocardioides dokdonensis*	TAS [19]
		Strain FR1436	TAS [19]
	Gram stain	Gram-positive	TAS [19]
	Cell shape	Rod	TAS [19]
	Motility	Non-motile	TAS [19]
	Sporulation	Nonsporulating	TAS [19]
	Temperature range	4 to 30 °C	TAS [19]
	Optimum temperature	25 °C	TAS [19]
	pH range; Optimum	5.0 to 10.0, 7.0	TAS [19]
	Carbon source	Adonitol, glycerol, melezitose, melibiose, ribose, sodium acetate, sodium citrate, sodium propionate, sodium pyruvate	TAS [19]
MIGS-6	Habitat	Sand sediment	TAS [19]
MIGS-6.3	Salinity	0 to 7% (*w*/*v*)	TAS [19]
MIGS-22	Oxygen requirement	Strictly aerobic	TAS [19]
MIGS-15	Biotic relationship	Free-living	TAS [19]
MIGS-14	Pathogenicity	Unknown	NAS
MIGS-4	Geographic location	Republic of Korea	TAS [19]
MIGS-5	Sample collection	2008	TAS [19]
MIGS-4.1	Latitude	37° 05′ N	TAS [19]
MIGS-4.2	Longitude	131° 13′ E	TAS [19]
MIGS-4.4	Altitude	Not reported	NAS

^a^Evidence codes - *IDA* Inferred from Direct Assay, *TAS* Traceable Author Statement (i.e., a direct report exists in the literature), *NAS* Non-traceable Author Statement (i.e., not directly observed for the living, isolated sample, but based on a generally accepted property for the species, or anecdotal evidence). These evidence codes are from the Gene Ontology project [44]

**Fig. 2:**
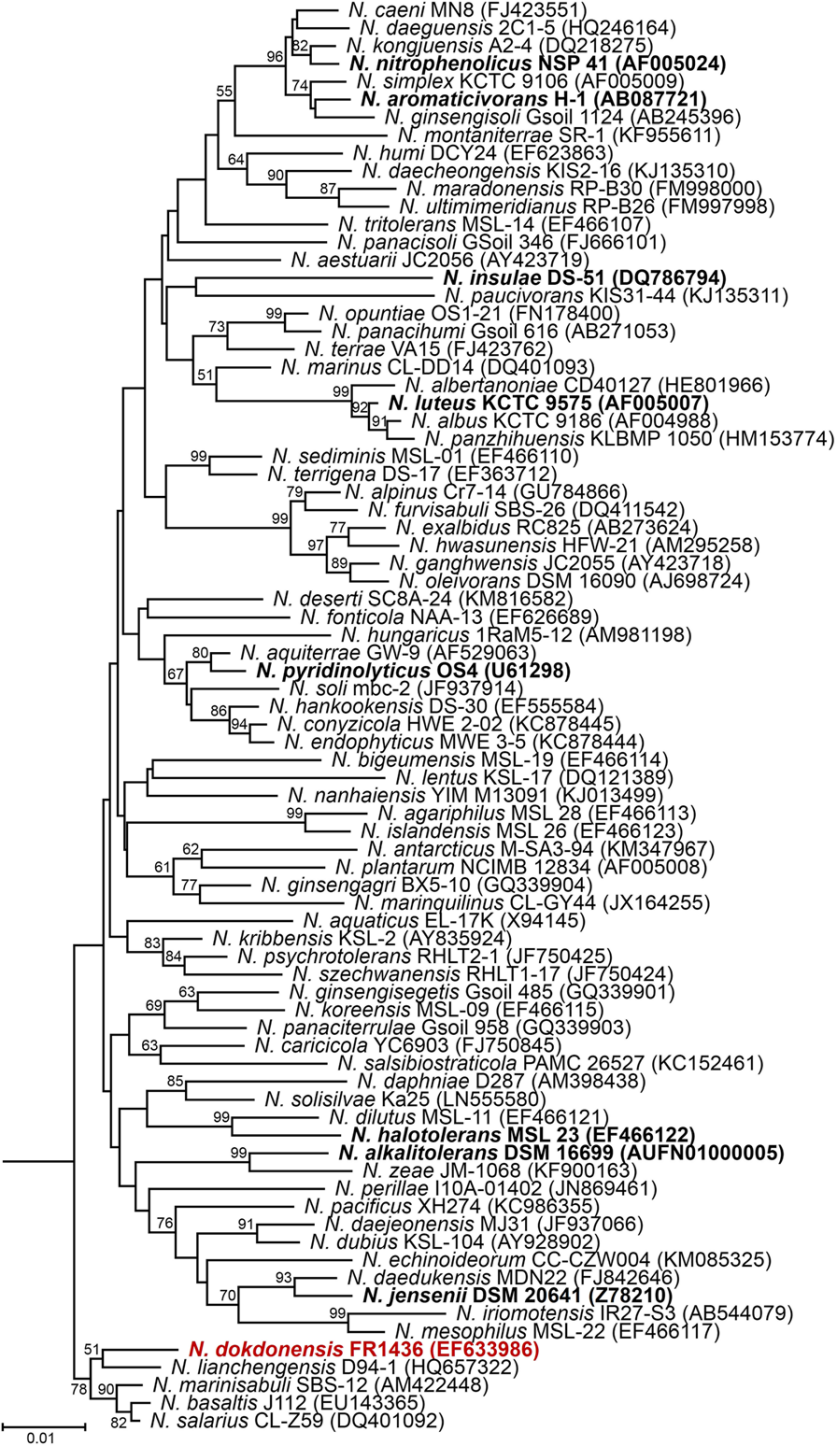
Phylogenetic relationship of the species in *Nocardioides*. A neighbor-joining tree based on the 16S rRNA gene was generated using MEGA 5 and Jukes-Cantor model was used for calculation of evolutionary distance based on the comparison of 1275 nucleotides. Bootstrap values (percentages of 1000 replications) greater than 50% are shown at each node; *Nocardia asteroids* NBRC 15531 (BAFO01000006) was used as an out-group. Scale bar represents 0.01 nucleotide substitutions per site. Accession numbers of the 16S rRNA gene are presented in the parentheses. Species for which genome sequences are available are indicated in bold

Phylogenetically, *N. dokdonensis* belongs to the family *Nocardioidaceae* of the order *Propionibacteriales*, and a phylogenetic tree based on the 16S rRNA genes of the type strains in the genus *Nocardioides* shows that *N. dokdonensis* FR1436 forms a sister clade with *N. lianchengensis* (Fig. 2), which was isolated from soil, and shares common ancestor with *N. marinisabuli*, *N. basaltis*, and *N. salaries*.

## Genome sequencing information

### Genome project history

As part of the project that investigates the genomic and metabolic features of bacterial isolates in and around Dokdo, the genome sequencing and analysis of *N. dokdonensis* FR1436 were performed at the Laboratory of Microbial Genomics and Systems/Synthetic Biology at Yonsei University. The complete genome sequence of *N. dokdonensis* FR1436^T^ (= KCTC 19309 ^T^ = JCM 14815 ^T^) has been deposited in GenBank under the accession number CP015079. The Bioproject accession number is PRJNA191956. A summary of the genome project is provided in Table 2.

**Table 2:** Project information

MIGS ID	Property	Term
MIGS-31	Finishing quality	Complete
MIGS-28	Libraries used	A 20-kb library
MIGS-29	Sequencing platforms	PacBio RS II system
MIGS-31.2	Fold coverage	355.4×
MIGS-30	Assemblers	SMRTpipe HGAP 3.0
MIGS-32	Gene calling method	Prokka
	Locus Tag	I601
	Genbank ID	CP015079
	Genbank Date of Release	March 31, 2016
	GOLD ID	Gp0037383
	BIOPROJECT	PRJNA191956
MIGS-13	Source Material Identifier	FR1436
	Project relevance	Environmental, soil bacterium

### Growth conditions and genomic DNA preparation

*N. dokdonensis* FR1436 was streaked on trypticase soy agar medium (Difco, 236,950) and incubated at 25 °C for 3 days. A single colony was inoculated in trypticase soy broth and incubated at 25 °C for 2 days. Cells in the exponential phase were harvested and genomic DNA was extracted using Wizard Genomic DNA Purification Kit (Promega, USA) according to the manufacturer’s protocol.

### Genome sequencing and assembly

Genome sequencing of *N. dokdonensis* FR1436 was performed using the PacBio RS II System (Macrogen, Inc., Republic of Korea). A 20-kb library and C4-P6 chemistry were used for the genome sequencing. A total of 200,435 continuous long reads and 1,551,246,448 base pairs were generated after genome sequencing and quality trimming of the sequencing reads. De novo assembly was conducted with SMRTpipe HGAP and scaffolding and gap filling were performed with SMRTpipe AHA. Finally, consensus sequences were generated with SMRTpipe Quiver.

### Genome annotation

Structural gene prediction and functional annotation were conducted using the Prokka program [22]. Additionally, we performed a functional assignment of the predicted protein-coding sequences using blastp against Pfam, Uniref90, KEGG, COG, and GenBank NR databases for more accurate annotation. tRNAscan-SE [23] and RNAmmer [24] were used for prediction of transfer RNAs and ribosomal RNAs, respectively. Assignment of the Clusters of Orthologous Groups was conducted with RPS-BLAST against COG database with an *e*-value cutoff of less than 1*e*-02. Clustered regularly interspaced short palindromic repeats were predicted with CRISPR Finder [25]. Proteins containing signal peptide and transmembrane helices were predicted using SignalP [26] and TMHMM [27], respectively. Secondary metabolite biosynthetic genes were predicted using AntiSMASH program [28].

## Genome properties

**Fig. 3:**
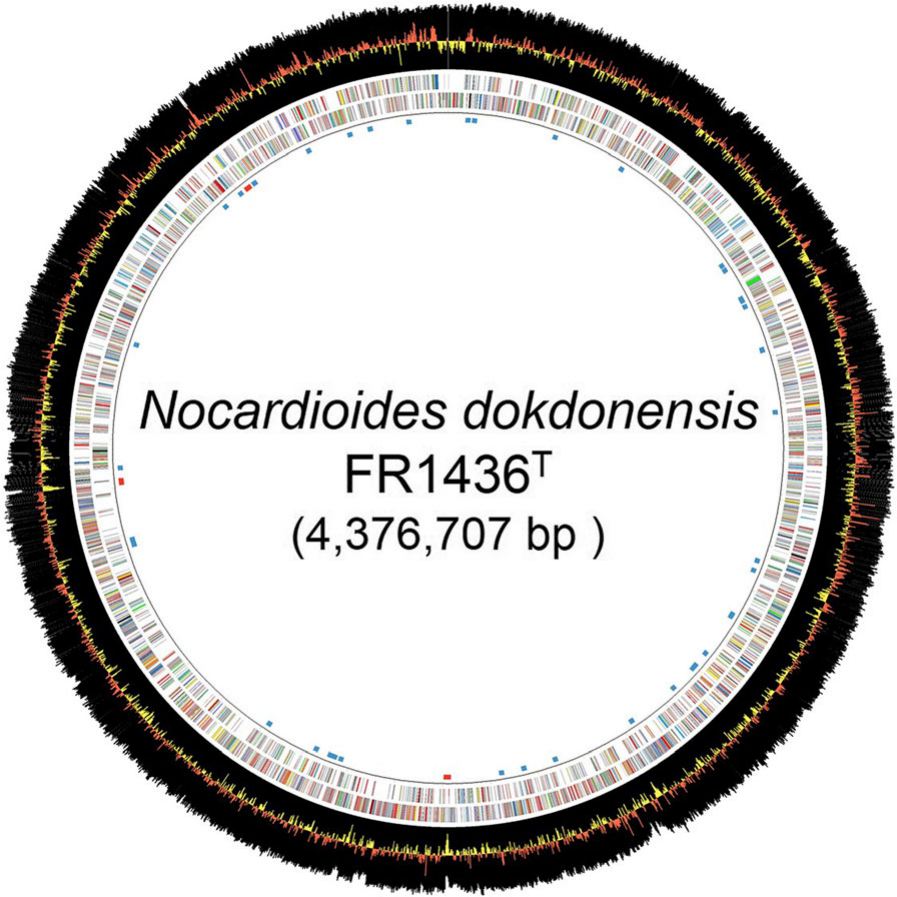
Circular representation of the genome of *N. dokdonensis* FR1436. The first and second circles from inside indicate COG-assigned genes in color codes. *Black* circle represents the G + C content and red-yellow circle is for the G + C skew. Innermost, *blue-scattered* spots are tRNA genes and red-scattered spots indicate rRNA genes

*N. dokdonensis* FR1436 has a single chromosome of 4,376,707 bp in length, and consists of 72.26% of G + C content (Fig. 3 and Table 3). The genome has 4165 genes that are comprised of 4104 CDSs, three rRNA operons, 51 tRNAs, and one tmRNA. Results from the analysis of KEGG pathways indicated that, in the genome of FR1436, all of the genes involved in glycolysis, gluconeogenesis, and citrate cycle are present and well conserved. Among the predicted genes, 71.38% of the genes were assigned putative functions and 2832 CDSs was functionally assigned to the COG categories (Table 4). Also in the genome, ten putative CRISPR repeats were predicted using the CRISPRFinder program, but there were no CRISPR-associated proteins next to the predicted repeat sequences. Two gene clusters, possibly associated with secondary metabolism, were predicted using the AntiSMASH program. One cluster (accession numbers ANH38050 to ANH38087) has genes associated with the phenylacetate catabolic pathway [29] and another cluster (accession numbers ANH40163 to ANH40204) has genes of type 3 polyketide synthases.

**Table 3:** Genome statistics

Attribute	Value	% of total
Genome size (bp)	4,376,707	100
DNA coding (bp)	4,059,326	92.75
DNA G + C (bp)	3,162,427	72.26
DNA scaffolds	1	
Total genes	4165	100
Protein coding genes	4104	98.54
RNA genes	61	1.46
Pseudogenes	0	0
Genes in internal clusters	ND*	ND*
Genes with function prediction	2973	71.38
Genes assigned to COGs	2832	69.01
Genes with Pfam domains	2584	62.04
Genes with signal peptides	343	8.24
Genes with transmembrane helices	1011	24.27
CRISPR repeats	10	10

*ND not determined

**Table 4:** Number of protein coding genes of *N. dokdonensis* FR1436 associated with the general COG functional categories

Code	Value	Percentage*	Description
J	151	3.68	Translation, ribosomal structure and biogenesis
A	1	0.02	RNA processing and modification
K	212	5.17	Transcription
L	164	4.00	Replication, recombination, and repair
B	1	0.02	Chromatin structure and dynamics
D	25	0.61	Cell cycle control, cell division and chromosome partitioning
V	41	1.00	Defense mechanisms
T	116	2.83	Signal transduction mechanisms
M	124	3.02	Cell wall/membrane/envelope biogenesis
N	3	0.07	Cell motility
U	29	0.71	Intracellular trafficking, and secretion
O	111	2.70	Posttranslational modification, protein turnover, chaperones
C	240	5.85	Energy production and conversion
G	145	3.53	Carbohydrate transport and metabolism
E	317	7.72	Amino acid transport and metabolism
F	75	1.83	Nucleotide transport and metabolism
H	106	2.58	Coenzyme metabolism
I	232	5.65	Lipid metabolism
P	133	3.24	Inorganic ion transport and metabolism
Q	86	2.10	Secondary metabolites biosynthesis, transport, and catabolism
R	321	7.82	General function prediction only
S	199	4.85	Function unknown
-	1272	30.99	Not in COGs

*The percentages are based on the total number of protein-coding genes in the genome

## Insights from the genome sequence

**Fig. 4:**
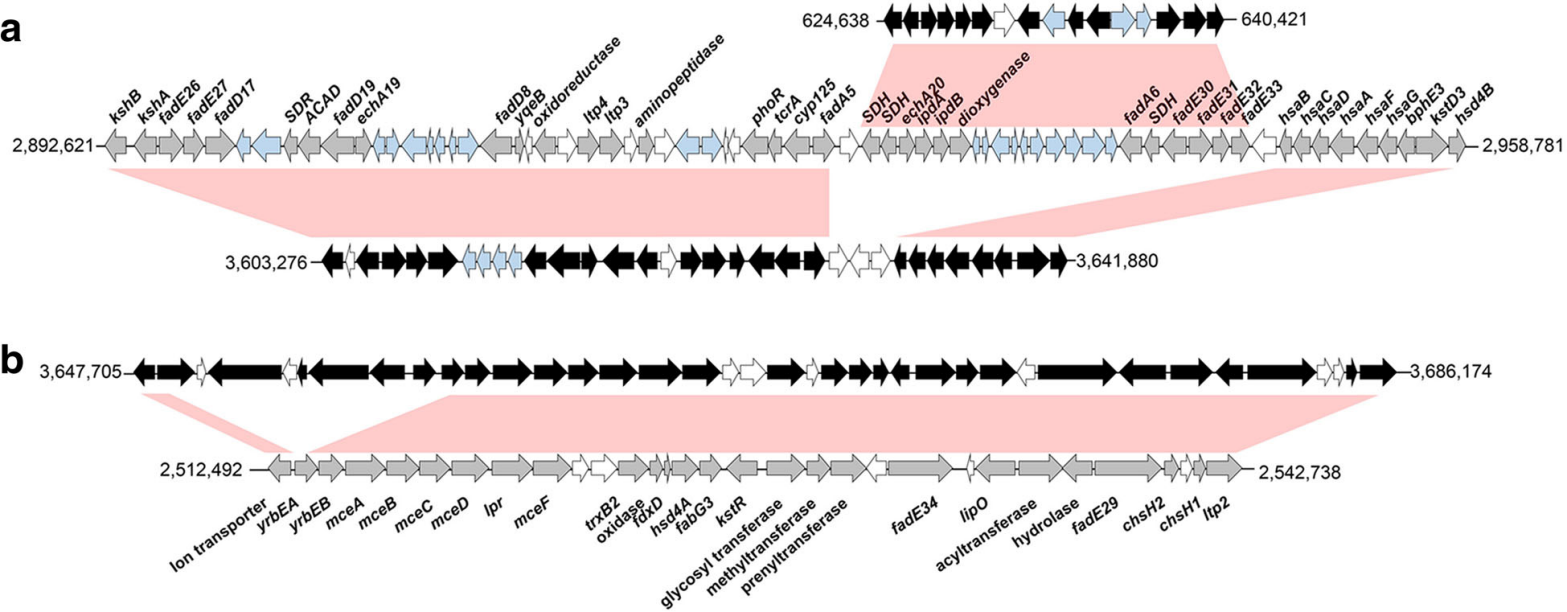
Steroid degrading gene clusters. Gene clusters were referred from the ones of *P. simplex* VKM Ac-2033D [35], for which genes associated with steroid degradation are indicated in grey arrows. Genes associated with steroid degradation in *N. dokdonensis* FR1436 are represented by *black arrows*. *Sky blue* indicates genes located in the cluster, but little information associated with steroid degradation. White arrows indicate genes encoding hypothetical protein. **a**. Gene cluster A involved in degradation of steroid ring A and B [35]. Accession numbers of the genes in *P. simplex* VKM Ac-2033D are AIY19941 to AIY17666. Accession numbers of the genes in *N. dokdonensis* FR1436 are ANH39848 to ANH39880 and ANH37060 to ANH37075. **b**. Gene cluster B involved in degradation of side chains of steroids [35]. Accession numbers of the genes are AIY19891 to AIY17347 for *P. simplex* VKM Ac-2033D and ANH39925 to ANH39888 for *N. dokdonensis* FR1436

**Fig. 5:**
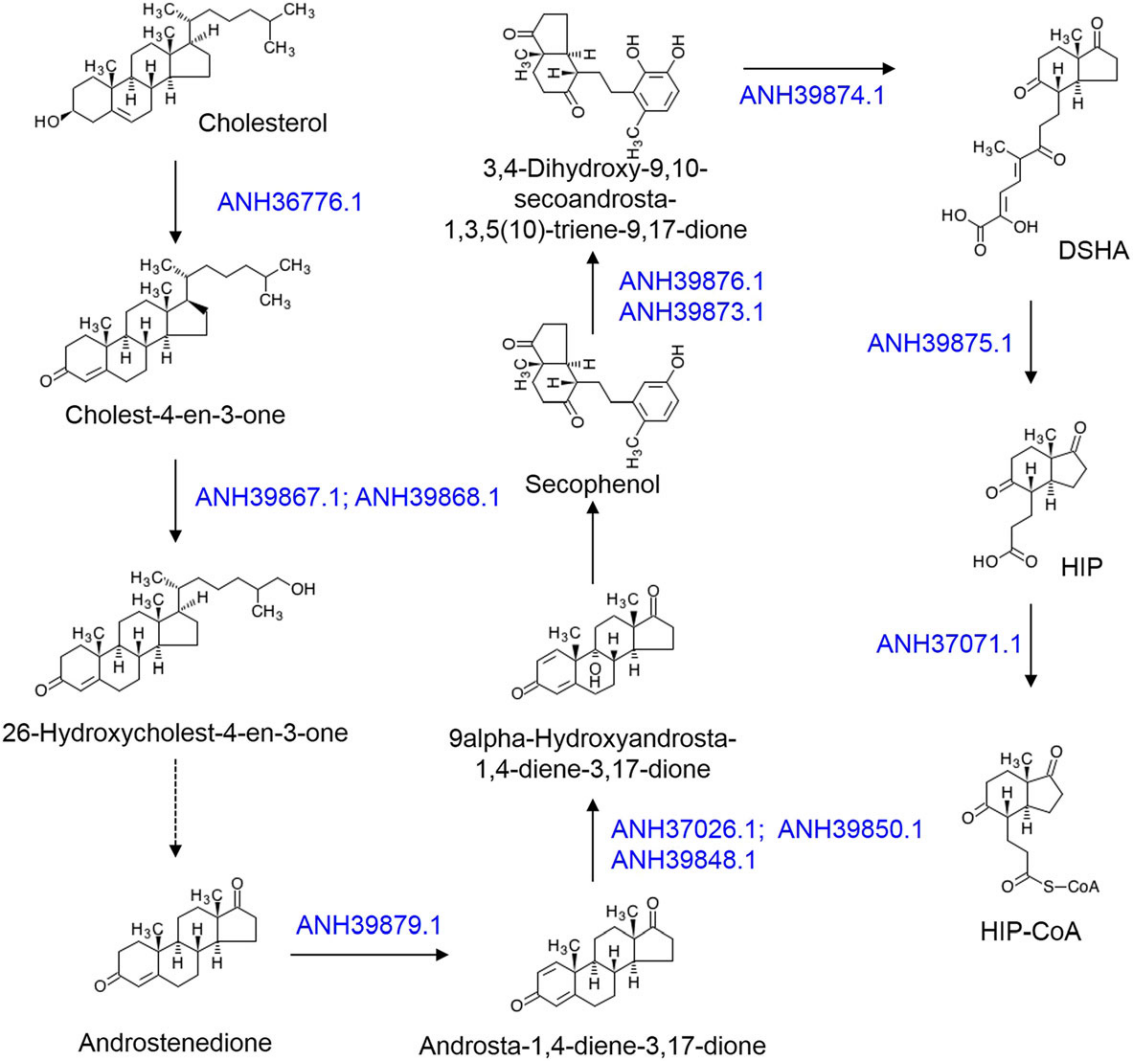
Cholesterol degradation pathway. Metabolic pathway was referred from the KEGG pathway map 00984. *Blue* indicates gene accession numbers involved in the cholesterol degradation in *N. dokdonensis* FR1436. DSHA, 3-hydroxy-5,9,17-trioxo-4,5:9,10-disecoandrosta-1(10),2-dien-4-oate; HIP, 9,17-dioxo-1,2,3,4,10,19-hexanorandrostan-5-oic acid

In the genome of *N. dokdonensis* FR1436, dozens of steroid-degrading genes were detected (Additional file 1). Major functions of steroids, essential biomolecules in living organisms, include maintaining membrane fluidity as a component of the cell membrane and controlling cell metabolism as signaling molecules [30]. Moreover, steroid medicines are used for treatment of a number of diseases from inflammation to cancer [31]. The molecular backbone of steroids is composed of three cyclohexanes and one cyclopentane. To the backbone, diverse side chains are attached to endow them with diverse functions [32]. Catabolic pathways of steroid degradation or modification have been analyzed in depth for some genera in the order *Corynebacteriales* [33–35]. In *Nocardioidaceae*, several large gene clusters, which have potential binding sites of the transcriptional regulator associated with steroid catabolism in their promoters, were predicted in the genome of *Pimelobacter simplex* VKM Ac-2033D [36]. In the genome of FR1436, gene cluster A, which is known to be involved in degrading steroid rings A/B, and gene cluster B, which is involved in degrading side chains, were detected (Fig. 4). However, in FR1436, cluster A is separated into two large gene clusters and an additional *mce* gene cluster, which is involved in steroid uptake [37], was detected (Additional file 1). In VKM Ac-2033D, cluster A is located approximately 350-kb downstream of cluster B, whereas in FR1436, cluster A is located 6 kb downstream. Moreover, two *kstR* and 11 *kstR2* genes, which encode the TetR family of transcriptional regulators and are reported to regulate cholesterol metabolism in mycobacteria [38], were detected (Additional file 1). Besides the genes in clusters A and B, genes encoding 3-beta-hydroxysteroid dehydrogenase (ANH36717 and ANH37882), 3-alpha-hydroxysteroid dehydrogenase (ANH37023 and ANH37488), and steroid delta-isomerase (ANH36955) were also detected in the genome of FR1436. Additionally, all genes involved in degradation of cholesterol to HIP-CoA were identified (Fig. 5). These results indicate that the genus *Nocardioides* can be useful for research and utilization of steroid metabolism.

## Conclusions

Steroids are important biomolecules in living organisms and carry out diverse roles as components of the cell membrane to signaling molecules [30]. Moreover, steroids are being used to treat various diseases from inflammation to cancer [31]. These indicate that research on modification of steroid compounds has infinite possibilities to improve human health. To date, studies on bacterial steroid metabolism have been mainly focused on the order *Corynebacteriales* [33–35]. Recently, genome analysis of the genus *Nocardioides* in the order *Propionibacteriales* revealed several kinds of gene clusters associated with steroid degradation [36]. In this study, we determined the complete genome sequence of *N. dokdonensis* FR1436 and analyzed the genome sequence to detect the presence of genes related to steroid metabolism. In the genome of FR1436, dozens of genes associated with steroid catabolism were detected in large gene clusters. These results demonstrate that bacteria in the genus *Nocardioides* can be used as promising candidates for steroid research and related fields of industry.

## Additional file

Additional file 1: Table S1. Genes associated with steroid metabolism. (XLSX 14 kb)

## Abbreviations

BLAST: Basic Local Alignment Search Tool
CDS: Coding sequence
COG: Clusters of Orthologous Groups
CRISPR: Clustered regularly interspaced short palindromic repeat
DSHA: 3-Hydroxy-5,9,17-trioxo-4,5:9,10-disecoandrosta-1(10),2-dien-4-oate
HIP: 9,17-Dioxo-1,2,3,4,10,19-hexanorandrostan-5-oic acid
KEGG: Kyoto Encyclopedia of Genes and Genomes
MIGS: Minimum information about a genome sequence
NR: Non-redundant
Pfam: Protein families
RPS-BLAST: Reversed position specific-BLAST
Uniref: UniProt reference clusters
